# Lessons learned from recruiting socioeconomically disadvantaged smokers into a pilot randomized controlled trial to explore the role of Exercise Assisted Reduction then Stop (EARS) smoking

**DOI:** 10.1186/1745-6215-16-1

**Published:** 2015-02-12

**Authors:** Tom P Thompson, Colin J Greaves, Richard Ayres, Paul Aveyard, Fiona C Warren, Richard Byng, Rod S Taylor, John L Campbell, Michael Ussher, Susan Michie, Robert West, Adrian H Taylor

**Affiliations:** Plymouth University Peninsula School of Medicine and Dentistry, ITTC Building, Plymouth Science Park, Plymouth, PL6 8BX UK; University of Exeter Medical School, St Luke’s Campus, Heavitree Road, Exeter, EX1 2LU UK; Nuffield Department of Primary Care Health Sciences, University of Oxford, New Radcliffe House, Walton Street, Jericho, Oxford OX2 6NW UK; Institute of Population Health Research, St George’s University of London, Cranmer Terrace, London, SW17 ORE UK; Research Department of Clinical, Educational and Health Psychology, University College London, 1-19 Torrington Place, London, WC1E 7HB UK; Department of Epidemiology and Public Health, Health Behaviour Research Centre, University College London, Gower Street, London, WC1E 6BT UK

**Keywords:** Harm reduction, Tobacco control, Physical activity, Recruitment, Smoking cessation, Primary care, Community medicine

## Abstract

**Background:**

Research is needed on what influences recruitment to smoking reduction trials, and how to increase their reach. The present study aimed to i) assess the feasibility of recruiting a disadvantaged population, ii) examine the effects of recruitment methods on participant characteristics, iii) identify resource requirements for different recruitment methods, and iv) to qualitatively assess the acceptability of recruitment. This was done as part of a pilot two-arm trial of the effectiveness of a novel behavioral support intervention focused on increasing physical activity and reducing smoking, among disadvantaged smokers not wishing to quit.

**Methods:**

Smokers were recruited through mailed invitations from three primary care practices (62 participants) and one National Health Stop Smoking Service (SSS) database (31 participants). Six other participants were recruited via a variety of other community-based approaches. Data were collected through questionnaires, field notes, work sampling, and databases. Chi-squared and t-tests were used to compare baseline characteristics of participants.

**Results:**

We randomized between 5.1 and 11.1% of those invited through primary care and SSS, with associated researcher time to recruit one participant varying from 18 to 157 minutes depending on time and intensity invested.Only six participants were recruited through a wide variety of other community-based approaches, with an associated researcher time of 469 minutes to recruit one participant. Targets for recruiting a disadvantaged population were met, with 91% of the sample in social classes C2 to E (NRS social grades, UK), and 41% indicating mental health problems. Those recruited from SSS were more likely to respond to an initial letter, had used cessation aids before, and had attempted to quit in the past year. Overall, initial responders were more likely to be physically active than those who were recruited via follow-up telephone calls. No other demographics or behaviour characteristics were associated with recruitment approach or intensity of effort. Qualitative feedback indicated that participants had been attracted by the prospect of support that focused on smoking reduction rather than abrupt quitting.

**Conclusions:**

Mailed invitations, and follow-up, from health professionals was an effective method of recruiting disadvantaged smokers into a trial of an exercise intervention to aid smoking reduction. Recruitment via community outreach approaches was largely ineffective.

**Trial registration:**

ISRCTN identifier: 13837944, registered on 6 July 2010

## Background

Smokers from disadvantaged groups (such as the unemployed, low-skilled manual workers, and people with mental health problems) attempt to quit at the same rate as others but the success rate in quitting is lower [1]. Smoking has been identified as one of the main contributing factors to health inequalities in industrial countries [2] and, in England and Wales, accounts for nearly half the difference of smoking-attributed mortality (among males) between the highest and lowest socioeconomic groups [3]. Smoking reduction may increase the motivation to quit, which is highly predictive of quit attempts, and reduce smoking dependence, which is related to successful quitting [4]. Also, offering an intervention to support smoking reduction may increase the reach of services to disadvantaged smokers who would not engage in cessation programs [5, 6]. Offering support for smoking reduction may attenuate the commonly reported barriers to engagement of ‘fear of failure’ and ‘fear of being judged’ when attempting to abruptly quit [7].

Evidence for the effectiveness of smoking cessation interventions for disadvantaged groups is limited [1, 8–10]. The lack of evidence may have resulted from both the inherent difficulties in recruiting and engaging with such groups in clinical trials, and the predominant focus on abrupt quitting rather than smoking reduction. Further research is therefore needed on how best to recruit disadvantaged smokers to increase intervention reach [7] with appropriate behavioral support [7, 10, 11]

More detailed and transparent information on the reach of trials and interventions (such as the proportion of the targeted population that participated) targeting disadvantaged groups is needed to better assess and plan interventions [12]. Various approaches may improve recruitment into studies among disadvantaged groups [13, 14], including the following: engagement with the target population when developing the intervention and preparing participant information about the study and intervention, using a variety of community networks and settings to invite the target population, and use of follow-up telephone calls to explain the study methods and intervention. In a review, the most effective strategies for recruiting smokers into trials [15] suggested that tailored interventions, recruitment methods that are proactive in nature (for example, approaching potential participants directly) [16], and more intensive recruitment strategies (such as repeated provision of information and contact) may help. But in general there is insufficient knowledge regarding the factors influencing recruitment and the most effective strategies for recruiting into randomized trials [17–19], particularly among disadvantaged groups.

This article reports on the feasibility and acceptability of strategies specifically designed to recruit disadvantaged smokers who wished to reduce their smoking but not quit, into a phase two pragmatic pilot randomized controlled trial: the Exercise Assisted Reduction then Stop (EARS) trial (Health Technology Assessment (HTA) number: 07/78/02, International Standardized Randomized Controlled Trial Number (ISRCTN): 13837944, UK Clinical Research Network (UKCRN) Study ID: 8937. The specific objectives of this study as related to the present paper were: i) to identify the feasibility of recruiting disadvantaged smokers through a variety of settings (through primary care, National Health Service (NHS) Stop Smoking Services (SSS), and through more generalized community approaches) as well as to examine the effect of using varying degrees of recruitment activity intensity (recruitment by invitation letter only compared with an invitation letter plus follow-up reminder telephone calls, and through various levels of community engagement); ii) to examine how recruitment of participants through different locations and different levels of recruitment intensity impact on participant characteristics; iii) to identify the time requirements associated with each recruitment approach; and iv) to qualitatively explore the effectiveness and acceptability of different approaches to recruitment.

Overall, the trial sought to identify uncertainties about the methods and intervention to support smoking reduction with physical activity and behavioral support. The two-arm randomized controlled trial consisted of a weekly one-to-one counselling-based intervention of up to 12 weeks, supporting self-directed changes in smoking and physical activity behaviors, compared with usual care (brief advice on available SSS), among disadvantaged smokers wishing to cut down but not quit. Data collection took place at baseline, eight weeks, and 16 weeks post baseline. The primary outcome was the number of participants achieving confirmed expired air carbon monoxide abstinence at four weeks post-quitting. Other outcomes included the number of quit attempts made, the number achieving at least 50% reduction in smoking at 16 weeks, changes in physical activity, and various other behavioral and process measures. The protocol and main findings of the study have been published [20].

## Methods

### Locating and defining a disadvantaged population

We set a target to recruit participants of whom at least 75% were unemployed or in social class C2 to E (NRS Social Grades, UK (skilled manual workers, semi-skilled and unskilled manual workers, state pensioners, casual and lowest grade workers, and those unemployed with state benefits only), 30% were single parents, and 20% had indicators of a mental health problem (indicated by answering ‘moderately’ or ‘extremely’ anxious or depressed to item five of the EQ-5D questionnaire). These criteria were based on the high prevalence of smoking among these groups [1]. Recruitment took place in the neighborhoods of Devonport and Stonehouse in Plymouth, United Kingdom, selected for having high deprivation (index of multiple deprivation score of 52 to 59.9, placing them within the 3% ‘most deprived’ areas in England [21]). Local data indicated that smoking prevalence among adults was over 40%, and the location had generally poor health, with a life expectancy 12.6 years lower than some other areas of the city [22].

### Inclusion and exclusion criteria

Participants were eligible if they were over 18-years-old, smoked at least 10 cigarettes per day (and had done so for at least two years), did not intend to quit in the next month, were able to engage in moderate intensity physical activity (walking without stopping for at least 15 minutes), were registered with a general practitioner (GP), and did not wish to use nicotine replacement therapy (NRT) to reduce smoking. The study focus was initially on reducing smoking, not quitting, so those who expressed a desire to quit immediately were referred directly to the NHS SSS without entering the study. Those wishing to use NRT were excluded to avoid any confounding of the effects of physical activity on their smoking behaviour. We excluded those with severe mental health problems and ongoing substance misuse due the potential difficulties of engaging them in the intervention, given the large uncertainties and complexities of its delivery, and those who may have put the safety of the research team at risk. Due to the exploratory nature of the study, participants were required to be able to converse in English.

### Recruitment

Recruitment was over a 12-month-period between May 2011 and May 2012, with a recruitment target of 120 smokers. Recruitment was split between two distinct approaches: 1) primary care and 2) other community-based approaches.

#### Primary care

Initially, one primary care medical practice in each of the two neighborhoods was identified and approached to be included in the research study; a third practice with patients from both areas was approached later in the study in order to expand the scope of recruitment to meet the planned sample size. We planned to recruit 50% (n = 60) of participants through primary care. GP practice lists were searched based on cursory inclusion and exclusion criteria (see Taylor *et al*., for more details [20]). A list of potential participants was generated and invitation letters, in batches of 100 per practice, were sent every two weeks from the GP with a postal reminder a week later. To begin with, postal invitations were sent without making any follow-up telephone calls so a response rate to a letter-only invitation could be established. Following on from this, and to increase reach to people with low literacy, telephone calls were made to those who did not respond directly to the invitation letter to check that they had received and understood the invitation, and to explore the effect of follow-up calls on increasing recruitment rates. If there was no answer on the first call a message was left to enquire if the invitation had been received and to leave a contact number for further information. Up to four more calls were made but, to avoid harassment, no further messages were left. Interested participants returned a form indicating interest in the study or telephoned a researcher. They were then screened for eligibility by telephone and provided consent for a researcher to contact their GP to confirm eligibility. Once eligibility was confirmed by their GP, the volunteer was invited to attend a baseline assessment.

#### Community

The other 50% (n = 60) of the targeted recruitment was categorized as ‘other community approaches’. There were two distinct recruitment methods. The first (for which a target of n = 30 was set) involved the searching of the local NHS SSS database for people who had used the service within the last two years but had failed to quit. The same procedure of mailed invitations and follow-up telephone calls as used for recruitment through GP practices was then adopted, without applying the cursory inclusion and exclusion criteria in the first instance. The second method involved outreach in an attempt to recruit smokers who may not engage with traditional services and may experience higher levels of disadvantage. Potential participants were contacted through: workplaces, educational sites, community sites, and a range of other media (see Table 1). Interested participants contacted the research team directly (in person) and indirectly by telephone or by returning contact details with a request for further information. Following screening, to determine eligibility, a time for attending a baseline session was arranged.

**Table 1:** **Locations and activities involved in community recruitment**

Workplace site	Recruitment activity
Local adult education and training provider	Flyers and information packs in the reception. Contact at centre informed about study and given packs to distribute.
Post Office Manual Data Entry Centre (MDEC)	Information cascaded through managers to all employees in team briefings.
Educational site	
Local primary school	Article in parent newsletter with study contact details.
Parent and toddler groups; several local children’s centers	Mother and toddler groups visited. Researchers attended groups and talked with parents. Posters and packs left, or given out during groups. Collected details of interested persons.
Community site or organization	
Job centre (Devonport)	Researcher outside the job centre approached smokers explaining the study; 100 packs and reply sheets given out over several periods in a week. Contact details of interested persons collected.
Local community hub cafe	Local health promotion sessions and food bank sessions attended by researchers; information given out to interested persons.
Local community cooperative organization	Flyers and posters given out to a local community employer for distribution.
YMCA (community-run gym)	Posters on display. Fitness Manager promoted study to users of the Stonehouse Gym.
	Researchers attended a children’s session; one pack given out.
Local gym	Gym instructors informed about study and provided with information packs and reply sheets to distribute to interested persons.
Local social club	Central contact informed about study and provided with information packs and reply sheets.
Public health	Posters and information packs with reply sheets given to the local health club in Devonport.
Three Local Housing Associations	180 flyers distributed through mailboxes in housing association residences in Plymouth; flyers distributed and attendance at residents’ meetings. Posters, flyers, and packs left at site for visitors.
Neighborhood Managers (City Council)	Researchers met with managers in Devonport and Stonehouse. Information distributed.
Local community learning centre	Information and flyers displayed. Researchers attended information sessions. Contact details of interested persons collected.
Other	
Local library	Flyers and posters on display.
Heart Radio/Plymouth Sound/Radio; local paper	Radio chat about the study and news advert in paper.
Word of mouth	First 60 trial participants asked to invite friends and/or acquaintances to join the study once they had completed the final follow-up assessment.
Individual contacts (for example, church minister, day support facility member, or publican)	Posters displayed by contacts.
Increasing Access to Psychological Therapies Service, Plymouth	Met and encouraged Psychological Wellbeing Practitioners to refer to the study opportunistically. Left flyers, information packs, and reply sheets to be distributed. Encouraged by email.
Posters displayed around local shops and businesses	Trial posters with contact details displayed in up to 50 local shops and businesses, (newsagents, hairdressers, tattoo parlors, and so on).

Informed consent was obtained from all successfully recruited participants before being randomized into the trial.

### Data collection

Data collection consisted of four elements:

1. Number of invitation letters sent, responses received, telephone calls made, participants declining participation, participants who were ineligible, and participants entering the trial through which recruitment location were all recorded on databases throughout the recruitment period, at the recruitment location and individual level, to allow conversion rates to be produced by recruitment location and by recruitment activity intensity.
2. As part of the main trial, the following data was collected at baseline: demographic information (age, sex, cohabiting status, cohabiting with other smokers, whether they were the parent of a resident child under 16, job status, age at leaving fulltime education, ethnicity, weight, and height), smoking history (age on starting smoking, longest period of cessation in the last year, attempts at cutting down, cessation aids used in past year, use of SSS, and satisfaction with previous use of SSS), number of cigarettes being smoked per day, Fagerström Test for Nicotine Dependence (FTND) [23, 24] scores, stage of readiness to use physical activity to control smoking behaviour (scored as either pre-contemplation, contemplation, preparation, action, or maintenance) [25], expired air carbon monoxide (CO) with cessation or abstinence defined as less than 10 parts per million (PPM) (Bedfont Smokerlyser, Harrietsham, Kent United Kingdom) which is deemed to represent biochemically verified abstinence [26], and physical activity data (self-reported seven day recall of physical activity and by accelerometer). In the present paper this data was used to compare the characteristics of those recruited through different locations and via different recruitment activity intensity.
3. The time spent by the research team on recruitment activity including: searching GP and SSS databases for potential participants (including screening potential participants for eligibility for GP databases only), preparation and mailing of invitation letters, making follow-up phone calls to non-responders to the mailed invitation, contacting interested participants to screen for eligibility, and arranging baseline appointments were recorded on the trial database. Additional information relating to the time spent recruiting through broader community approaches was recorded via researcher activity logs, diaries, and work sampling procedures.
4. Qualitative data were collected through a combination of field notes, regular documented meetings, audio recorded interviews with the research team, and opportunistic feedback from stakeholders. Semi-structured, audio recorded interviews were also completed with the use of a topic guide on a purposively sampled range of participants after completing the trial to cover a range of demographics and achieved outcomes.

### Data analyses

To calculate the conversion rates from invitation to entry into the trial, a percentage was derived from the total number of invites sent out via each location and the resulting number of randomizations from each location. Conversion rates for broader community approaches was not possible to determine due to the open-ended nature of the majority of the methods (it is unknown how many people may have read, for example, a flyer or poster and therefore it is impossible to derive a denominator).

Pearson’s chi-squared and t-tests (independent, two-sample, and two-tailed) were completed for categorical and continuous variables, respectively, to compare characteristics of those recruited through primary care and SSS, and to compare those recruited by initial invitation letter only or by initial invitation letter plus follow-up telephone calls. Statistical analyses were completed using Stata SE (version 12.0, StataCorp LP, College Station, Texas, USA).

To calculate time associated with various recruitment methods, the time associated with samples of invitations sent to groups of potential participants sent via each location which received the same intensity of effort (considered to represent best practice) was totalled and divided by the number of participants successfully recruited. All time associated with broader community approaches was also totalled and divided by the number of participants successfully recruited via these approaches. This resulted in a total amount of time spent by the research team per participant randomized. Reasons for ineligibility were also recorded.

Qualitative data were analyzed using surface-level thematic analyses and case studies in NVivo (version 9 (QSR International Pty Ltd, Doncaster, Victoria, Australia). Ethical approval for the study was granted by the NHS National Research Ethics Service Committee South West, in the United Kingdom (Local Research Ethics Committee number: 10/H0106/59).

## Results

### Recruitment rates

Invitations sent from primary care (with no follow-up telephone calls, n = 361) led to 5.1% of those invited being randomized into the study. With attempted contact by follow-up telephone calls to non-responders (n = 485) this proportion increased to 8.8%. Invitations sent from SSS (with no follow-up telephone calls, n = 255) led to 6.8% of those invited being randomized into the study. With follow-up telephone calls to non-responders (n = 137) this proportion increased to 11.1%.

### Comparison of participant characteristics

Baseline descriptive data for the recruited sample can be found in Table 2. Those recruited through SSS, compared with primary care, were more likely to be recruited through letter invitation (*X*^2^ (1, *N =* 93) = 6.43, *P* = 0.01), to have used cessation aids before (*X*^2^ (1, *N =* 93) = 26.35, *P* <0.01), and to have made a quit attempt in the past year (*X*^2^ (1, *N =* 93) =8.23, *P* <0.01). No other variables were associated with recruitment from primary care or SSS in univariate analyses.

**Table 2:** **Sample characteristics**

	Total sample (N = 99)
Female (n, (%))	55 (56.1)
Age (mean (SD); median (IQR))	46.6 (11.3); 47.5 (38.3 - 55.4)
Ethnicity (n, (%))	
White British	95 (96.0)
Cohabiting (n, (%))	50 (50.5)
Children under 16 (n, (%))	28 (28.3)
Single parent^a^ (n, (%))	6 (6.1)
Employed (n, (%))	54 (54.5)
Job status^b^ (n, (%))	
A to C1	9 (9.0)
C2 to E (excluding unemployed)	45 (45.5)
Unemployed	45 (45.5)
Age on leaving education (mean (SD); median (IQR))	16.3 (1.9); 16 (15 - 16)
Age on starting smoking (mean (SD); median (IQR))	14.7 (3.5); 14 (13 - 16)
Does partner or other cohabitant smoke? (n, (%))	
Yes	31 (31.3)
No	27 (27.3)
Not applicable	41 (41.4)
BMI (mean (SD), n; median (IQR))	28.1 (6.4), 98; 27.3 (22.4 - 32.4)
Indicated mental health problem^c^ (n, (%))	41 (41.4)
Duration of smoking (years, mean (SD); median (IQR))	31.9 (12.2); 34.2 (23.3 - 42.2)
Previously used SSS (n, (%))	41 (41.4)
Satisfaction with previous use of SSS (if used) (scale 1 to 11); mean (SD), n	8.3 (2.8), 40
Participant made a quit attempt lasting 24 hours or more in the past year (n, (%))	37 (37.4)
Did the participant cut down before previous cessation?^d^ (n, (%))	
Yes	5 (13.5)
No	32 (86.5)
Total, n	37
Used cessation aids as part of a quit attempt in previous 12 months^e^ (n, (%))	
Yes	29 (78.4)
No	8 (21.6)
Total, n	37
Used cessation aids not as part of a quit attempt in previous 12 months (n, (%))	
Yes	21 (33.9)
No	41 (66.1)
Total, n	62
Self-reported cigarettes smoked per day (mean (SD); median (IQR))	21.6 (14.3), 19.1 (14.4 - 24.4)
Expired air CO (ppm), mean (SD)	18.0 (8.0)
FTND (mean (SD); median (IQR))	5.6 (2.0); 6 (4 - 7)
Readiness to use physical activity as a way of controlling smoking, action and maintenance stage (n, (%))	9 (9.1)
Self-reported minutes of moderate and vigorous physical activity over previous 7 days (median (IQR))	315 (120 - 540)
Accelerometer data	n = 66
Minutes spent in moderate/vigorous/very vigorous activity per day (mean (SD), n; median (IQR))	31.9 (24.5); 28.37 (13.2 - 44.8)
Step counts (mean (SD), n; median (IQR))	7701.7 (3536.2); 7343.5 (4909 - 9853)

^a^All single parents female apart from one male, recruited through SSS. As a percentage of women (up to aged 47, the oldest parent with an under 16 year child) the percentage of female single parents across all recruitment methods was 17%.

^b^Job status from NRS social grades, UK.

^c^Answered ‘moderately’ or ‘extremely’ anxious or depressed to item five of the EQ-5D questionnaire.

^d^Includes only smokers who had stopped smoking for at least 24 hours in the previous year.

^e^At least 24 hours’ reported abstinence.

BMI: Body mass index; CO: Carbon monoxide; FTND: Fagerström test for nicotine dependence; IQR: Interquartile range; ppm: Parts per million; SD: Standard deviation; SSS, Stop Smoking Service.

Those recruited via initial invitation letter, compared with those recruited via a follow-up telephone call, were more likely to have used SSS in the past (*X*^2^ (1, *N =* 93) = 4.45, *P* =0.035) and to self-report completing at least 30 minutes of moderate and vigorous physical activity per day at baseline (*X*^2^ (1, *N =* 92) = 4.45, *P* =0.035), but other variables were not associated with the recruitment method.

### Recruitment rates and associated researcher time based on best practice

Based on the figures above and data collected on researcher time dedicated to each recruitment method, to recruit 100 participants (with a 5.1% conversion rate) through primary care (via *letter invitation only,* without follow-up) would require 1,961 invitations to be sent and 1,800 minutes (30 hours) of researcher time. To recruit 100 participants via letter invitation and follow-up telephone reminders (8.8% conversion rate) would require 1,336 invitations to be sent and would require 7,134 minutes (118.9 hours) of researcher time.

To recruit 100 participants (with a 6.8% conversion rate) through SSS (via *letter invitation only,* without follow-up) would require 1,471 invitations to be sent and 2,400 minutes (40 hours) of researcher time. To recruit 100 participants via letter invitation and follow-up telephone reminders (11.1% conversion rate) would require 901 invitations to be sent and 7,547 minutes (125.8 hours) of researcher time. Further details can be found in Tables 3 and 4.

**Table 3:** **Time associated with recruiting 100 participants through stop smoking services**

	Denominator		To recruit 100 (letter only - 6.8% response)		To recruit 100 (letter plus follow-up telephone calls - 11.1% response)	
Activity	Number	Time (minutes)	Number	Time (minutes)	Number	Time (minutes)
Database searching	Per practice/location	60	Per practice	60	Per practice	60
Initial screening	To produce 200 eligible	60	1,471	441	901	270
Mailing invitations	200	240	1,471	1,765	901	1,081
GP screening of responses	1	2	100	200	100	200
Associated researcher time	1	24*	100	2,400	100	7,547
		157**				

*Letter only. **Letter plus follow-up telephone call. GP: General practitioner.

**Table 4:** **Time associated with recruiting 100 participants through primary care**

	Denominator		To recruit 100 (letter only - 5.1% response)		To recruit 100 (letter plus follow-up telephone calls - 8.8% response)	
Activity	Number	Time (minutes)	Number	Time (minutes)	Number	Time (minutes)
Database searching	Per practice/location	60	Per practice	60	Per practice	60
Initial screening	To produce 200 eligible	60	1,961	588	1,336	401
Mailing invitations	200	240	1,961	2,353	1,336	1,603
GP screening of responses	1	2	100	200	100	200
Associated researcher time	1	18*	100	1,800	100	7,134
		145**				

*Letter only. **Letter plus follow-up telephone call. GP: general practitioner.

**Figure 1:**
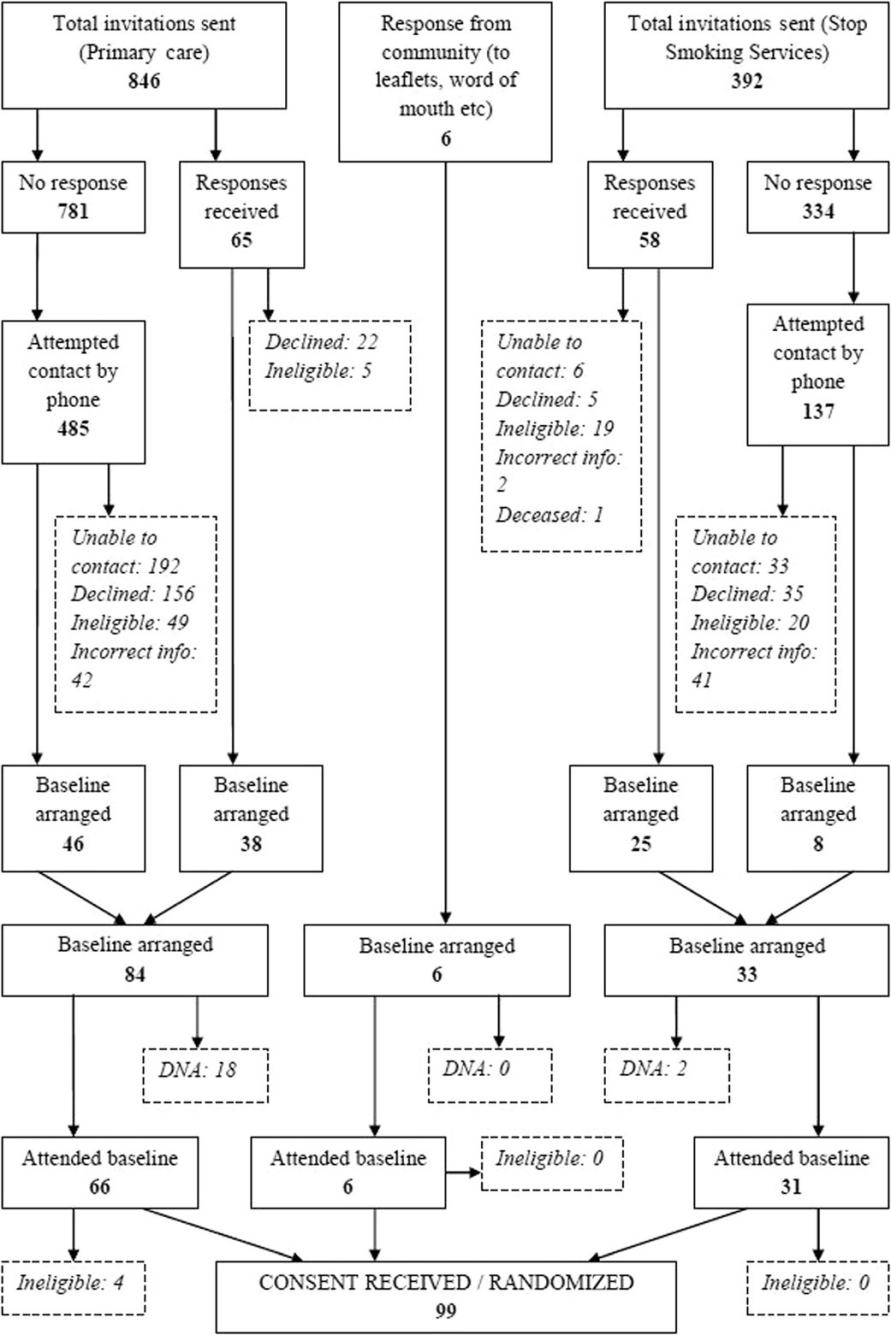
**CONSORT diagram showing recruitment approaches and participant flow up to randomization.**

The reasons for ineligibility were similar across recruitment methods (Table 5). The main reason for ineligibility (for more than 50% of those ineligible) was due to the individual having already quit smoking. A summary of recruitment via different locations is shown in Table 6, and the flow of participants through each recruitment method up to randomization is shown in Figure 1.

**Table 5:** **Reasons for ineligibility (other community not shown, 0% ineligible)**

Reasons for ineligibility	Primary care	SSS
Health/physical (%)	15.8	20.5
Already quit (%)	57.9	53.8
Smokes <10 cigarettes per day (%)	10.5	10.4
Close friend or relative of somebody already in the trial (%)	0.0	5.1
Currently using NRT (%)	5.3	5.1
Under 18 years (%)	0.0	5.1
Wants to quit immediately (%)	10.5	0.0

NRT: Nicotine replacement therapy; SSS, Stop Smoking Services.

**Table 6:** **Participant recruitment by recruitment method**

Recruitment method	N = 99, n (%)	% of target
Primary care	62 (62.6)	62/60 (103.3%)
Letter only	31 (31.3)	
Letter plus reminder telephone calls	31 (31.3)	
Stop Smoking Services	31 (31.3)	31/30 (103.3%)
Letter only	24 (24.2)	
Letter plus reminder telephone calls	7 (7.1)	
Community (without invitation letter)	6 (6.1)	6/30 (20.0%)

### Qualitative observations

A qualitative summary of the variety of other community approaches to recruitment are presented in Table 7. The various approaches resulted in only six participants entering the study and had a directly associated researcher time of 469 minutes to recruit one participant. To recruit 100 participants via other community approaches would require 46,900 minutes (781.7 hours) of researcher time.

**Table 7:** **Location and summary of effectiveness of recruitment efforts**

Workplace site	Relative success and qualitative observations
Local adult education and training provider	Total recruited = 0. Despite being followed-up on after initial provision of information, nobody came forward expressing an interest in the study. Location was identified as an attempt to target the unemployed and low skilled. A general feeling that the information became lost amongst lots of other available information.
Post Office Manual Data Entry Centre (MDEC)	Total recruited = 0. After initial meeting and briefing with the personnel manager, information was distributed at team meetings to all employees (n = 500. Despite following up with the personnel manager, nobody came forward expressing an interest. No confirmation of the quality of information that was cascaded to all employees; uncertainty over how well or enthusiastically the information was distributed. Likely to have been a low priority among the managers and a potential burden on their time.
Educational site	
Local primary school	Total recruited = 0. A small article about the study published and distributed to parents within the newsletter failed to attract any interest. Potentially intended to target single parents, but likely to be too broad an approach which people took little notice of as the information became lost amongst other more relevant information in the newsletter. Potentially out of place in the school letter context.
Mother and toddler groups; several local children’s centers	Total recruited = 0. Intended to target single parents as much as possible, the mother and toddler groups consisted of relatively low numbers, not all of whom were smokers. Small amounts of interest were shown, but researchers reported that the mothers’ focus was on their children and they were generally not very receptive to the information being offered. Researcher potentially viewed as an ‘outsider’.
Community site/organization	
Job centre (Devonport)	Total recruited = 1. Intended to target the unemployed, one person was recruited into the trial from approximately 100 information packs being distributed. Researcher found it to be quite an ‘intrusive’ activity on people smoking outside the job centre and met with some degree of hostility. Reported a sense that people would take the information just to get them to ‘go away’. A feeling that people were not very receptive to the information as they were there for other reasons with other pressing concerns. Potentially being viewed as ‘an outsider’.
Local community hub cafe	Total recruited = 0. Intended to target the unemployed and low skilled. Researchers reported a feeling that the people attending this location had multiple other serious issues (housing, drug addiction, and so on) which made them unreceptive to the information on offer. For most, smoking behaviour was not a high priority.
Local community cooperative organization	Total recruited = 0. Reports that once the information had been handed over and staff briefed about the study, it would quickly become a low priority among staff given information for distribution.
YMCA (community-run gym)	Total recruited = 0. No idea on the number of smokers actually using the service. Potential again for the enthusiasm for promoting the study to be lost once the information is left with those outside the study team, despite follow-up attempts.
Local gym	Total recruited = 0. Impression that promoting the study was a very low priority for the gym instructors, with no interest being generated.
Local social club	Total recruited = 2. The contact at the small local social club was very proactive and involved with the study. They had their own motivation to promote healthy initiatives to the local community and as such generated interest. The comparative success of this location was reported to be solely due to the individual’s motivation for promoting the study and encouraging their service users to take part.
Public health	Total recruited = 0. Similar reports to other groups where information was left for groups attended by potential participants - not all attending were smokers, and with relatively low numbers attending, no interest was generated.
Three Local Housing Associations	Total recruited = 0. Potential for information to be dropped to houses which had already received an invitation via their GP. This type of invitation possibly lacked the ‘authority’ of the invitation coming directly from their GP.
Neighborhood Managers (City Council)	Total recruited = 0. It was again reported that whilst enthusiasm was high amongst the neighborhood managers when meeting with the research team, the study took a very low priority for what is a very busy work force.
Local community learning centre	Total recruited = 0. Intended to target the unemployed and low skilled, it was unpredictable how many people would attend the sessions at which the researchers provided information and again not all attendees would be smokers. Potentially seen as ‘an outsider’.
Other	
Local library	Total recruited = 0. No way of knowing how many people read or saw the information on display. Potential for information to become lost amongst swathes of other information.
Heart Radio/Plymouth Sound/Radio Devon/Newspaper	Total recruited = 0. Broad awareness of the study was generated and interest attracted from people too far outside the study areas to be offered inclusion. The approach was not targeted enough at the disadvantaged groups intended.
Word of mouth	Total recruited = 0. Proved to be ineffective, attracting no interest. Potentially due to lack of motivation on an individual level in promoting the study; potentially could be improved by incentivizing referral.
Individual contacts (such as minister of religion, local day support facility member, and publican)	Total recruited = 1. One person recruited opportunistically through a researcher’s local contact. Relatively small reach via this approach and again reliant on individual promotion of study by people outside the study team.
Increasing Access to Psychological Therapies Service (IAPT), Plymouth	Total recruited = 0. Intended to target those with mental health problems. Systems for recruiting and referring individuals were problematic and at times convoluted (due to data protection). Communication between the research team and IAPT was difficult as there was a sense that the study was a low priority for the practitioners who had other issues to deal with.
Posters displayed around local shops and businesses	Total recruited = 0. Generally reported to be wholly ineffectual, assumed to be due to individuals’ lack of motivation to take the initiative and contact the research team directly.

One participant was recruited opportunistically after enquiring at the health centre where the study was based about support services for smokers; another participant was recruited through a friend who had been approached via SSS. A summary of the characteristics of the participants interviewed at the end of the study can be found in Table 8.

**Table 8:** **Characteristics of participants who were interviewed at the end of the study**

	Control (N = 10) (20% of sample)	Intervention (N = 15) (30% of initial sample)
Demographics		
Age (years); mean, *(*SD)*)*	46 (11)	52 (11)
Gender (m*:*f)	5:5	8:7
Job status^*a*^; n (%)		
A1 to C1	0 (0)	0 (0)
C2 to E (excluding unemployed)	5 (50)	9 (60)
Unemployed	5 (50)	6 (40)
Single parenthood; n (%)	1 (10)	0 (0)
Baseline data		
Smoking characteristics		
Tobacco (grams/day); mean (SD)	17.4 (8.7)	18.4 (12.5)
FTND; mean (SD)	5.3 (1.9)	4.9 (1.7)
Self-reported MVPA (minutes/week); mean (SD)	595 (757)	401 (644)
Self-reported MVPA (minutes/week); Range; n (%)		
0	2 (20)	4 (27)
1 - 499	4 (40)	9 (60)
500 - 1999	3 (30)	1 (7)
2000+	1 (10)	1 (7)
Quit attempt in past year; n (%)	5 (50)	5 (33)
Outcomes		
Quit attempt made; n (%)	0 (0)	5 (33)
*Four-*week CO confirmed abstinence; n (%)	n/a	4 (80)
>50% reduction in smoking; n (%)	2 (20)	4 (>33)*
No change in smoking; n (%)	8 (80)	4 (>26)*
Recruitment		
Avenue (GP:SSS:community); n (%)	5:5:0 (50:50:0)	7:6:2 (47:40:13)
Type (letter:telephone:other); n (%)	7:3:0 (70:30:0)	6:7:2 (40:47:13)

^a^Job status from NRS social grades, UK.

*no data on 50% reduction for three participants. CO: Carbon monoxide; FTND: Fagerström test for nicotine dependence; ppm: Parts per million; MVPA: Moderate and vigorous physical activity; SD: Standard deviation.

The novel approach of actively promoting support for reduction in smoking was well received by the majority of the sample. Many emphasized the appeal of reduction against the alternative of stopping abruptly. The appeal of reducing smoking appeared to stem from an underlying desire to change behaviour, but due to a lack of confidence or desire to stop abruptly, reduction seemed a much more manageable objective:

‘I think that was probably it, the reduction thinking. Well you know, rather than sort of go cold turkey and completely stop I thought, “Oh you know, you could help me reduce it,” which you did, so you know that obviously it worked.’ *(Female, 60 to 65 years, unemployed, moderate smoker, intervention)*

‘Yeah it did as well, because I thought, “I don’t really want to, I am not ready to stop yet,” and I thought cutting down is quite good.’ *(Female, 35 to 40 years, employed part-time, moderate smoker, control)*

For some, past experiences of failed quitting heightened the appeal of support for reduction as a novel approach to tackling their smoking behaviour:

‘Well, for three or four years I’ve been trying to give up smoking [and] last month [I] done 10 months, and then I had a smoke…This one appealed to me because you cut down, you know, every week you cut down two cigarettes a week and you just cut down and cut down and I eventually got down to none.’ *(Male, 60 to 65 years, unemployed, moderate smoker, intervention, successful quitter)*

The message in the trial invitation of support to cut down did not appear to threaten people’s sense of control over their own behaviour, compared with a message around abrupt quitting. For some, it was clear that a pervading message of the need to stop smoking would have completely alienated them from engaging in the study:

‘[The researcher] was saying “We can help you to cut down. We may in time be able to stop you smoking” and I said “Well, that’s a very sensible attitude to take” because someone telling me “I’m going to stop you smoking” I’d tell them to… go away! So that’s what made me do it initially, because they weren’t threatening me that they could stop me smoking. But even at this time, there is no-one that can tell me “I can stop you smoking” you know what I mean?’ *(Male, 55 to 60 years, unemployed, heavy smoker, intervention)*

The invitation was designed, as was the intervention, to be supportive and client-centered and a step away from traditional services, and the supportive and unpressurised nature of the invitation was well received:

‘You know, I say, when [the researcher] gave me the leaflet I thought “Yeah, alright, I’ve heard all this before” and I thought “Well, here we go with the hard sell”. But [they were] totally different. [They were] so relaxed, so friendly, and that’s what pushed me towards it. If [they] had tried to come across with the hard sell I would most probably have just ignored [them] and said cheerio. But I think just approaching people in a friendly manner…I mean, sometimes it helps.’ *(Male, 55 to 60 years, employed full time, heavy smoker, intervention)*

Negative experiences of using NRT and other medicinal therapies emerged as a strong theme linked to motivation for taking part. The intervention was envisaged as a novel alternative to NRT, and people’s description of past experiences seemed to confirm this particular aspect of the intervention:

‘Oh I’ve tried a couple of times to cut out smoking totally ‘cause I’ve tried the smoking aids and all the things you know, the puffer and the patches and that hasn’t worked, so I thought “Oh well, I’ll give this a try then try and cut down,” yeah.’ *(Male, 40 to 45 years, employed part-time, very heavy smoker, intervention)*

The appeal of the invitation to reduce smoking, as opposed to quit, was supported by the Health Trainers when interviewed, who identified a desire to reduce smoking as the primary factor when asked ‘What attracted participants to the study?’:

‘They’re only coming in because we’ve said reducing smoking, rather than quitting and I think that’s what is getting them into the surgery…’ *(Health Trainer three)*

‘Um, a different approach maybe, slightly different to what they’ve actually done before, and I think it wasn’t about quitting, it’s about trying to reduce rather than them quitting…’ *(Health Trainer two)*

Overall, there was a clear indication that the invitation appealed to, and reached, people who would not have been interested in support to quit.

## Discussion

This unique study provides much needed data on the engagement of disadvantaged populations with a focus on harm reduction as opposed to abrupt quitting, and provides a systematic attempt to assess the effects of increasing intensity of recruitment activity (via follow-up telephone calls) and recruitment methods on accessing disadvantaged groups.

Recruitment targets were met for the operational definition of a disadvantaged group of 91% in social class C2 to E (target 75%), 41% with an indicated mental health problem (target 20%), but failed to reach the proposed target of 30% for single parents (17% of sample). This was a particularly difficult group to target, partly due to this information not being available from GP and SSS databases, and also due to the difficulty in targeting single parents within the community. From the attempts that were made to target this sample within the community (parent and toddler groups and school settings) no single parents were recruited. More robust and effective methods are needed to understand the best way to engage with single parents who smoke. Since conducting the study we have become aware of several smoking and harm reduction studies (for example, studies cited in the National Institute for Health and Care Excellence (NICE) guidelines on harm reduction, 2013, and these may provide further ideas on how best to recruit single parents. Future ideas for recruitment of single parents may include the use of midwifery records which record the smoking status of antenatal women.

The greatest reason (>50%) for ineligibility was an individual having already quit smoking, although it is possible that potential participants used this as an excuse for not participating. Records of smoking status, held by both GP practices and SSS, were obviously dated in terms of the last contact at which they were recorded as smoking, thereby increasing the resources needed for inviting and screening.

The sample consisted overall of relatively heavy smokers (with a mean of 20 self-reported cigarettes per day, compared with a national average of 12.7 cigarettes per day) [27], indicating that the invitation to support reduction can recruit heavier smokers, building on previous survey data [28]. The sample self-reported high levels of daily physical activity, averaging more than the recommended 30 minutes of moderate andvigorous activity per day, although objectively measured physical activity suggested that individuals were overestimating their levels of activity. There is evidence to suggest that this is fairly typical of a disadvantaged population due to higher levels of activity associated with work and active transport [29–31]. The higher levels of activity may also reflect self-selection bias; the trial invitation referred to an intervention which included physical activity and lifestyle support which may have attracted a more active sample. Of those recruited, the gender balance was relatively even and similar to levels of engagement within the NHS SSS [32]. Overall, approximately 44% and 56% of all mailed invitations went to males and females respectively, indicating very similar recruitment rates for males (7.7%) and females (7.4%), suggesting the approach to recruitment and appeal of the invitation is equally effective at recruiting both males and females.

Whilst the use of follow-up telephone calls was effective at increasing recruitment rates, this increased researcher time (and costs) about five to seven-fold per participant recruited. The findings suggest that those recruited via follow-up telephone calls represented a harder-to-reach population, being both less likely to have used SSS in the past and less physically active. The use of follow-up telephone calls therefore may offer added value in reaching the more service-resistant smokers. It is possible that the reach of the intervention was higher than indicated in the recruitment rates presented; potentially anywhere between 10 and 66% of smokers invited into the study were not interested or were ineligible due to having a desire to quit [33]. For example, if 1,000 invites were sent, but potentially 66% of those invited were not part of the targeted population, the conversion rates of 5.1 to 11.1% presented here may in fact represent 11.6 to 25.2%, allowing for those who were no part of the intended targeted population for recruitment.

Differences were observed in those recruited through primary care and SSS predominantly in terms of smoking history. Those recruited through SSS were more likely to have used cessation aids in the past and more likely to have made a quit attempt in the previous 12 months. Another significant difference was that those invited through SSS were more likely to respond directly to the letter invitation than to the follow-up telephone call. These differences probably reflected a much more motivated group coming through SSS, as they had already engaged with a service to help address their smoking behaviour and were likely to have contemplated changing smoking behaviour. Of those recruited through primary care nearly three quarters (72.6%) had never previously engaged with SSS. When considered alongside differences in previous cessation aid use and previous quit attempts, this finding suggests that those recruited via primary care represented a much harder-to-reach group of smokers than those who had attended SSS.

The use of an invitation to receive support to cut down, sent out via primary care, could therefore be a valid way of increasing the reach of traditional smoking services. The nature of the invitation itself (not being branded as part of the SSS and offering support to cut down as opposed to quit) may also have played a large part in increasing the interest in participation in the study among those harder-to-reach smokers, and qualitative data supported this premise. There appears to be value in the invitation as a successful ‘smoking cessation induction approach for those who would not otherwise engage with traditional services.

Other community approaches were generally very unsuccessful, only recruiting six out of a targeted 30 participants. Data collected suggested this was due to three main reasons. First, when information was given to third parties for distribution the effectiveness relied on the individual’s motivation and priority for promoting the study, which was frequently low. Second, when the researchers took an active role in promoting the study in community-based locations they felt they were viewed as an ‘outsider’ and treated with some degree of skepticism (as has been shown elsewhere [13]) and, on occasion, hostility. Third, the recruitment approach of distributing information in the form of flyers, posters, and public advertisements, all of which relied on the smoker’s motivation to directly contact the research team, was shown to be completely ineffective. An additional element restricting the effectiveness of the community-based recruitment approaches was concern over the researchers’ personal safety, which restricted the kinds of activity that were deemed appropriate. Our experiences mirrored those of public health outreach workers trying to recruit smokers into SSS to attempt abrupt quitting in the same location a year before the present trial.

Overall, more research is needed about community-based recruitment approaches, although the indications from the present study suggest they are likely to be ineffective. Additional time spent with, and incentives for, community groups could be explored along with enrolling community ‘gatekeepers’ into the study as part of the research team and promoting partnership working. There is little evidence to suggest that the distribution of information via posters and other community media is effective; the possibility remains that doing so may raise the profile and increase the legitimacy of the research, making recruitment via other avenues more likely [34], although this cannot be quantified from the current research.

It is a limitation that the present study did not seek to recruit homeless and other disadvantaged smokers who were not registered with a GP, as well as excluding those with serious mental illness (groups demonstrating high levels of smoking), but within the scope of the study this was necessary to ensure adequate screening during recruitment and safety of the research team. Smoking reduction trials may have particular appeal amongst such groups and future research in this area would be appropriate.

Overall, assessing the reach of the current study (in line with the RE-AIM framework, used for assessing an intervention’s Reach, Effectiveness, Adoption, Implementation, and Maintenance [35]) is problematic due to lack of any denominator figure for the eligible population from which the sample was derived. Ethical approval was not obtained for profiling those who were invited into the study but did not respond and were not successfully contacted by the research team. A future study should carefully consider ways to address this in order to more firmly establish the reach of the study in terms of accessing the most disadvantaged groups within society.

## Conclusions

This study has demonstrated that using General Practice lists is a powerful way of recruiting patients to health interventions in an area of deprivation, and much more effective than community approaches. More than 98% of people in the United Kingdom in all areas are registered with a GP and on average visit 5.3 times per year [36]. People trust information provided via their GP more than other sources [37], and this almost certainly was the reason for the much higher response rate. It provides important and pragmatic information for the future recruitment of disadvantaged populations into research trials.

## Abbreviations

BMI: Body mass index
CO: Carbon monoxide
EARS: Exercise assisted reduction then stop
FTND: Fagerström test for nicotine dependence
GP: General practitioner
HTA: Health technology assessment
IQR: Interquartile range
ISRCTN: International Standardized Randomized Controlled Trial Number
LREC: Local Research Ethics Committee
NHS: National Health Service
NICE: National institute for health and care excellence
NRT: Nicotine replacement therapy
PA: Physical activity
PC: Primary care
SD: Standard deviation
SSS: Stop smoking services.
